# The Influence of Probiotic* Lactobacillus casei* in Combination with Prebiotic Inulin on the Antioxidant Capacity of Human Plasma

**DOI:** 10.1155/2016/1340903

**Published:** 2016-03-14

**Authors:** Paulina Kleniewska, Arkadiusz Hoffmann, Ewa Pniewska, Rafał Pawliczak

**Affiliations:** Department of Immunopathology, Faculty of Biomedical Sciences and Postgraduate Training, Medical University of Lodz, Ulica Zeligowskiego 7/9, 90-752 Lodz, Poland

## Abstract

The aim of the present study was to assess whether probiotic bacteria* Lactobacillus casei* (4 × 10^8^ CFU) influences the antioxidant properties of human plasma when combined with prebiotic Inulin (400 mg). Experiments were carried out on healthy volunteers (*n* = 32). Volunteers were divided according to sex (16 male and 16 female) and randomly assigned to synbiotic and control groups. Blood samples were collected before synbiotic supplementation and after 7 weeks, at the end of the study. Catalase (CAT), superoxide dismutase (SOD), glutathione peroxidase (GPx) activity, and the ferric reducing ability of plasma (FRAP) in human plasma were examined. The administration of synbiotics containing* L. casei* plus Inulin resulted in a significant increase in FRAP values (*p* = 0.00008) and CAT activity (*p* = 0.02) and an insignificant increase in SOD and GPx activity compared to controls. Synbiotics containing* L. casei* (4 × 10^8^ CFU) with prebiotic Inulin (400 mg) may have a positive influence on human plasma antioxidant capacity and the activity of selected antioxidant enzymes.

## 1. Introduction

Reactive oxygen species (ROS) are reactive chemical individuals incorporating oxygen atoms with an unpaired electron (radical) or O-O bond and are capable of participating in chemical reactions [1]. ROS play a crucial role in the basic biological processes in the body. These molecules participate in oxidation and reduction reactions (in the respiratory chain) and the removal of toxins, renew energy (ATP), and enable oxygen transport by hemoglobin, activation of cytochrome P450, and the phagocytosis of microorganisms. However, their overproduction can lead to free radical reactions, resulting in damage to lipids, proteins, carbohydrates, and nucleic acids. The quick and easy diffusion of ROS and their ability to react with multiple nonspecific components cells may lead to disturbances of many biological processes, which may result in the development of a number of diseases.

There are two types of ROS sources: endogenous and exogenous. Endogenous sources comprise those generated intracellularly and acting within the cell, and these include free radicals formed within the cell and then released into the surrounding area. Exogenous sources of ROS include a large number of chemical pollutants, medications, principally cancer chemotherapeutics, environmental agents (like nongenotoxic carcinogens), various xenobiotics, particularly chlorinated ones, metal ions, tobacco smoke, barbiturates, or radiation [2, 3]. ROS can also be divided into oxygen-centered nonradicals (singlet oxygen, ^1^O_2_; hydrogen peroxide, H_2_O_2_; ozon, O_3_) and oxygen-centered radicals (hydroxyl radical, ^•^OH; superoxide anion, O_2_^•−^; or hydroperoxyl, HO_2_^•^) [4].

Fortunately, the human organism has developed a number of enzymatic and nonenzymatic defense mechanisms against the harmful effects of ROS. Antioxidant enzymes, such as superoxide dismutase (SOD), catalase (CAT), and glutathione peroxidase (GPx), play an important role in the prevention of free radical damage in living organisms. Their mechanism of action creates an integrated system of antioxidant protection [5].

The biological role of SOD catalyzes the reaction of O_2_^•−^ dismutation. The enzyme is found in three forms: copper-zinc SOD (Cu/ZnSOD, SOD1), manganese SOD (MnSOD, SOD2), and extracellular superoxide dismutase (EC-SOD, SOD3) [6]. SOD1 is present in the cytosol and in the mitochondrial intermembrane space [7, 8]. SOD2 is localized in all mammalian cells but mainly in the mitochondrion and peroxisomes. The dismutation of O_2_^•−^ by MnSOD is similar to the reaction conducted by SOD1 and consists of two stages. MnSOD activity can be induced by a number of factors, including interleukin-1, liposaccharides (LPS), and TNF-*α*, and its increased activity protects cells against oxidative stress [6, 9]. SOD3 is present in plasma, tissues, and extracellular fluids such as synovial fluid and lymph.

Another important antioxidant enzyme which closely interacts with SOD is CAT. This enzyme decomposes H_2_O_2_ to O_2_ and H_2_O and is present in most organisms exposed to O_2_. CAT acts as both a catalase and a peroxidase. While its basic function is catalytic, in the presence of low H_2_O_2_ concentrations, it can also oxidize low molecular weight alcohols such as methanol or ethanol to aldehydes and water [10, 11]. CAT activity depends on substrate concentration, pH, temperature, inhibitors, or activators. The absence of CAT in the blood and other tissues in the human body leads to the occurrence of acatalasia, which is manifested by recurrent inflammation of the gums and ulcers in the mouth, or hypocatalasia. Reduced CAT activity is visible in a number of diseases accompanied by oxidative stress and inflammation, including atherosclerosis, pneumonia, tuberculosis, hepatitis, type 2 diabetes, cancer, or neurodegenerative diseases [12].

The main function of GPx is to reduce H_2_O_2_ and organic peroxides with the involvement of reduced glutathione (GSH). This enzyme is capable of restoring GSH using NADPH. Human GPx has several isozymes and each of them is encoded by a different gene. GPx isozymes are characterized by various features including structure, function, reactivity, substrate specificity, or location. GPx exists in eight isoforms, the most important of which are cytosolic (GPx-1, cGPx), gastrointestinal (GPx-2, giGPx), plasma (GPx-3, pGPx), and the phospholipid hydroperoxides peroxidase (GPx-4, phGPx) located in the cytoplasm and mitochondria. The products of lipid peroxidation (LO) may have an effect on GPx, one particular example being 4-hydroxynonenal (4-HNE), which reduces the amount of GSHt and inactivates cGPx by reaction with -SH group [13].

The World Health Organization regards probiotics as living microorganisms which have beneficial health effects if they are administered in appropriate amounts. Gibson and Roberfroid [14] define prebiotics as “nondigestible food ingredients that beneficially affect the host by selectively stimulating the growth and/or activity of one or a limited number of bacteria in the colon.” Finally, the term “synbiotic” is used when a product contains both probiotics and prebiotics.

In recent years, the number of studies, both* in vitro* and* in vivo*, related to the antioxidant properties of probiotics has significantly increased [15, 16]. The aim of the present study was to assess whether the probiotic bacteria* L. casei* influences the antioxidant properties of human plasma when administered in combination with prebiotic Inulin.

## 2. Materials and Methods

### 2.1. Experimental Design

This randomized study was carried out at the Medical University of Lodz (Poland) from December 2014 to February 2015. The total number of healthy volunteers (aged 20–35) was first divided into groups of men (*n* = 16) and women (*n* = 16). The subjects were then randomly assigned to synbiotic and control groups. Only subjects who had given voluntary informed consent were included in the study. The exclusion criteria included medical conditions requiring drug therapy, administration of nonsteroidal or anti-inflammatory drugs, use of any antimicrobial agent within the previous month, history of gastrointestinal diseases, acute infections, or food allergy. Also, athletes, subjects who were consuming yogurts at the time, subjects who were on special diets which might affect the antioxidant properties of plasma, or subject who were taking antioxidant vitamins were excluded. Blood samples were collected from the forearm veins before synbiotic supplementation and after seven weeks, at the end of the study.

The experimental procedures were approved by the Ethical Committee of the Medical University of Lodz (number RNN/801/14/KB).

### 2.2. Tested Dietary Supplement

A synbiotic supplement containing* Lactobacillus casei* with Inulin was purchased from ICN Polfa Rzeszow S.A., Poland. Each capsule contained 4 × 10^8^ CFU lyophilized* Lactobacillus casei* and 400 mg of Inulin. One capsule of synbiotic per day was administered to subjects, at dinner time for 7 weeks. The control group did not consume any synbiotics during the time of the study.

### 2.3. Preparation of Samples

Blood was collected into two tubes containing appropriate anticoagulant (EDTA or heparin). Blood samples were drawn from forearm veins into Vacuette tubes with EDTA as an anticoagulant for the CAT, SOD, and GPx assays or heparin as an anticoagulant for the FRAP assay. The blood was centrifuged at 1000 ×g for 10 minutes at 4°C or at 3000 ×g for 10–15 minutes at 4°C (Hettich Universal 320R Centrifuge). The obtained plasma was collected into Eppendorf tubes, frozen (−80°C), and stored for further examinations. Unfrozen, previously prepared plasma was used for measurement of antioxidant enzymes and FRAP.

### 2.4. Measurement of Plasma Antioxidant Capacity by FRAP Method

To determine the reducing ability of plasma, the FRAP method was used [17]. FRAP is a method to evaluate enzymatic and nonenzymatic plasma antioxidants such as low molecular weight peptides like bilirubin, C and E vitamins, uric acid, and albumins. The test works on the principle that the antioxidants contained in a sample reduce ferric-tripyridyltriazine Fe^3+^-TPTZ to the ferrous form Fe^2+^-TPTZ, which absorbs light at 593 nm.

*Chemicals*. The following chemicals were used: acetate buffer (300 mM; pH = 3,6); TPTZ (2,3,5-triphenyltetrazolium chloride; 10 mM); hydrochloric acid (40 mM); ferric chloride (20 mM). All reagents were purchased from Sigma-Aldrich (Ulica Szelagowska 30, 61-626), Poland.

*Assay Protocol*. The reaction solution was prepared by mixing 10 volumes of 300 mM acetate buffer, pH 3.6 with 1 volume of 10 mM TPTZ in 40 mM HCl and 1 volume of 20 mM FeCl_3_·6H_2_O. Following this, 30 *μ*L of human plasma and 90 *μ*L of deionized water were added to 900 *μ*L of the FRAP reagent. Absorbance was measured at 593 nm with a spectrophotometer (Perkin-Elmer Lambda 25).

### 2.5. Measurement of CAT Activity in Human Plasma

To measure CAT activity, a Catalase Assay Kit (Item number 707002), manufactured by Cayman Chemical Company, Ann Arbor, MI (BIOKOM, Ulica Wspolna 3, 05-090 Janki, Poland), was used. This method is based on the reaction of an enzyme with methanol in the presence of optimal H_2_O_2_ concentration. The produced formaldehyde is measured colorimetrically with 4-amino-3-hydrazino-5-mercapto-1,2,4-triazole (Purpald) as chromogen. Purpald specifically forms a bicyclic heterocycle with aldehydes, which, upon oxidation, changes from colorless to purple [18].

*Chemicals*. Catalase Assay Kit, Item number 707002, consisted of Catalase Assay Buffer (2 mL of Catalase Assay Buffer diluted with 18 mL of HPLC-grade water), Catalase Sample Buffer (5 mL of Catalase Sample Buffer diluted with 45 mL of HPLC-grade water), catalase formaldehyde standard (4,25 M formaldehyde); Catalase-Control (powder was dissolved in 2 mL of final Sample Buffer; subsequently, 100 *μ*L of reconstituted enzyme was diluted with 1.9 mL of final Sample Buffer), catalase potassium hydroxide (4 mL of 10 M potassium hydroxide), Catalase Hydrogen Peroxide (40 *μ*L of 8.82 M Catalase Hydrogen Peroxide was diluted with 9.96 mL of HPLC-grade water), Catalase Purpald (4 mL of 4-amino-3-hydrazino-5-mercapto-1,2,4-triazole in 0.5 M hydrochloric acid), catalase potassium periodate (1.5 mL of potassium periodate in 0.5 M potassium hydroxide), methanol, and HPLC-grade water.

*Assay Protocol*. After preparation of CAT Standard wells, each well on the plate was filled with 100 *μ*L of diluted Assay Buffer, 30 *μ*L of methanol, and 20 *μ*L of sample (plasma). Following this, 20 *μ*L of hydrogen peroxide was added to all used wells, and the plate was covered and incubated on a shaker for 20 minutes at room temperature (Thermo Scientific MaxQ 4000). To terminate the reaction, 30 *μ*L of potassium hydroxide was added. After a 10-minute incubation on the shaker with Catalase Purpald (30 *μ*L per well), the solution was finally incubated with potassium periodate (5 minutes, 10 *μ*L per well). Absorbance was read at 540 nm.

### 2.6. Measurement of SOD Activity in Human Plasma

To measure SOD activity, a Superoxide Dismutase Assay Kit (Item number 706002), manufactured by Cayman Chemical Company, Ann Arbor, MI (BIOKOM, Ulica Wspolna 3, 05-090 Janki, Poland), was used. This method uses tetrazolium salt for detection of superoxide radicals generated by hypoxanthine and Xanthine Oxidase. This assay measures all three types of SOD [19].

*Chemicals.* The Superoxide Dismutase Assay Kit (Item number 706002) consisted of the following: Assay Buffer (3 mL of Assay Buffer diluted with 27 mL of HPLC-grade water), Sample Buffer (2 mL of Sample Buffer diluted with 18 mL of HPLC-grade water), Radical Detector (50 *μ*L of Radical Detector diluted with 19.05 mL of diluted Assay Buffer), SOD Standard (100 *μ*L of bovine erythrocyte SOD), Xanthine Oxidase (50 *μ*L of Xanthine Oxidase diluted with 1.95 mL of diluted Assay Buffer), and HPLC-grade water.

*Assay Protocol.* After preparation of SOD Standard wells, each well on the plate was filled with 200 *μ*L of diluted Radical Detector and 10 *μ*L of sample (plasma). To initiate the reaction, 20 *μ*L of diluted Xanthine Oxidase was added to all used wells; the plate was mixed for a few seconds, covered, and incubated on a shaker for 30 minutes at room temperature (Thermo Scientific MaxQ 4000). Absorbance was read at 440–460 nm with a plate reader (Tecan Sunrise with software Magellan Standard).

### 2.7. Measurement of GPx Activity in Human Plasma

To measure GPx activity, the Glutathione Peroxidase Assay Kit (Item number 703102), manufactured by Cayman Chemical Company, Ann Arbor, MI (BIOKOM, Ulica Wspolna 3, 05-090 Janki, Poland), was used. In this method, GPx activity is indirectly measured by a coupled reaction with GR. GSSG, produced upon reduction of H_2_O_2_ by GPx, becomes recycled to its reduced state by GR and NADPH. Oxidation of NADPH to NADP+ is related with decrease in absorbance at 340 nm [20].

*Chemicals.* Glutathione Peroxidase Assay Kit Item number 703102 consisted of GPx Assay Buffer (3 mL of GPx Assay Buffer was diluted with 27 mL of HPLC-grade water), GPx Sample Buffer (2 mL of Sample Buffer was diluted with 18 mL of HPLC-grade water), glutathione peroxidase-Control (10 *μ*L of glutathione peroxidase was diluted with 490 *μ*L of final Sample Buffer), GPx Cosubstrate Mixture (each vial was reconstituted with 6 mL of HPLC-grade water and mixed), and GPx Cumene Hydroperoxide.

*Assay Protocol.* After preparation of the background wells and positive control wells, the sample wells were prepared: 100 *μ*L of final Assay Buffer, 50 *μ*L of GPx Cosubstrate Mixture, and 20 *μ*L of sample (plasma) were added. To initiate the reaction, 20 *μ*L of GPx Cumene Hydroperoxide was added to all used wells and the plate was carefully mixed for a few seconds. Absorbance was read every minute at 340 nm with a plate reader to obtain readings at five time points.

### 2.8. Statistical Analysis

All results were expressed as mean ± standard error of the mean (SEM). Groups were compared using the Student's *t*-test and Mann-Whitney rank sum test. The selection of appropriate tests depended on the distribution of the obtained data. *p* values lower than 0.05 were considered statistically significant.

## 3. Results

### 3.1. Evaluation of FRAP Values

**Figure 1:**
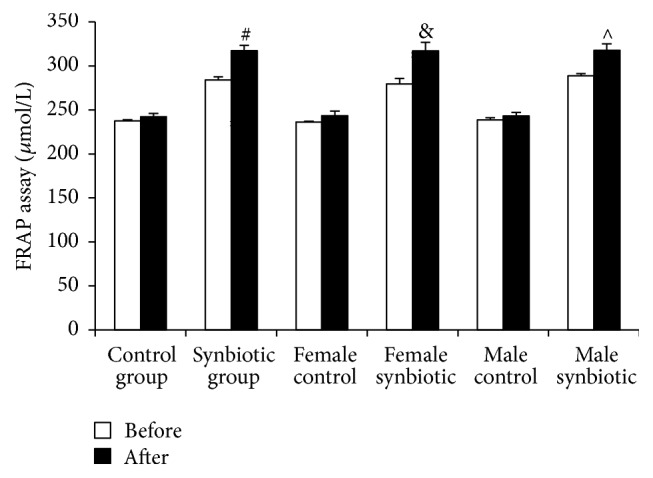
The influence of combination of probiotic* Lactobacillus casei* (4 × 10^8^ CFU) and prebiotic Inulin (400 mg) on the FRAP values. Data is shown as mean ± SEM. ^#^ *p* = 0.00008; ^&^ *p* = 0.008; ^∧^ *p* = 0.004* versus* control group.

The FRAP assay revealed a nonsignificant increase from 237.44 ± 1.37 *μ*mol/L to 242.34 ± 3.24 *μ*mol/L (*p* = 0.19) in the control group but a significant increase from 284.01 ± 3.64 *μ*mol/L to 317.32 ± 6.08 *μ*mol/L (*p* = 0.00008) in the synbiotic group. In the female-control group, antioxidant capacity increased insignificantly from 236.28 ± 0.98 *μ*mol/L to 241.53 ± 5.00 *μ*mol/L (*p* = 0.35), while, in the female-synbiotic group, plasma antioxidant capacity rose significantly from 279.41 ± 6.31 *μ*mol/L to 317.03 ± 9.62 *μ*mol/L after synbiotic administration (*p* = 0.008). In the male-control group, antioxidant capacity increased insignificantly from 238.61 ± 2.49 *μ*mol/L to 243.16 ± 4.08 *μ*mol/L (*p* = 0.39), while, in the male-synbiotic group, plasma antioxidant capacity rose significantly from 288.60 ± 2.79 *μ*mol/L to 317.60 ± 7.45 *μ*mol/L following synbiotic administration (*p* = 0.004) (Figure 1).

### 3.2. Evaluation of CAT Activity

**Figure 2:**
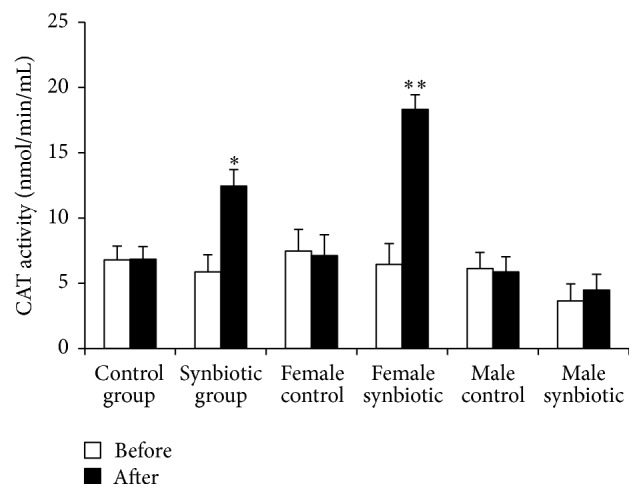
The influence of combination of probiotic* Lactobacillus casei* (4 × 10^8^ CFU) and prebiotic Inulin (400 mg) on the CAT activity. Data is shown as mean ± SEM. ^*∗*^ *p* = 0.02; ^*∗∗*^ *p* = 0.007* versus* control group.

CAT activity was found to increase insignificantly from 6.79 ± 1.07 nmol/min/mL to 6.85 ± 0.97 nmol/min/mL in the control group. However, CAT activity showed a significant (*p* = 0.02) increase from 5.87 ± 1.31 nmol/min/mL to 12.45 ± 1.26 nmol/min/mL over the course of the experiment in the synbiotic group. In the female-control group, CAT activity decreased from 7.46 ± 1.68 nmol/min/mL to 7.12 ± 1.59 nmol/min/mL. In contrast, CAT activity was significantly higher in the female-synbiotic group and increased from 6.44 ± 1.6 nmol/min/mL to 18.32 ± 1.12 nmol/min/mL over the seven weeks of the study (*p* = 0.007). In the male-control group, CAT activity decreased from 6.13 ± 1.24 nmol/min/mL to 5.87 ± 1.16 nmol/min/mL. In the male-synbiotic group, the initial value of CAT activity was 3.65 ± 1.29 nmol/min/mL. After synbiotic administration, CAT activity increased insignificantly to 4.48 ± 1.22 nmol/min/mL (*p* = 0.7) (Figure 2).

### 3.3. Evaluation of SOD Activity

**Figure 3:**
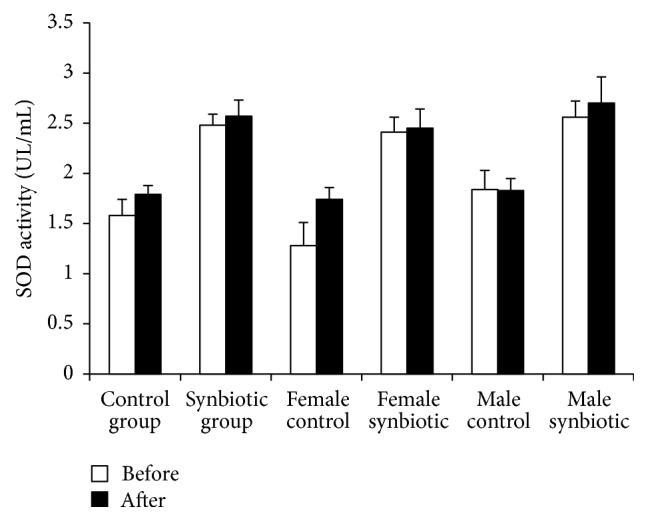
The influence of combination of probiotic* Lactobacillus casei* (4 × 10^8^ CFU) and prebiotic Inulin (400 mg) on the SOD activity. Data is shown as mean ± SEM.

In the control group, SOD activity demonstrated a nonsignificant increase from 1.58 ± 0.16 U/mL to 1.79 ± 0.09 U/mL (*p* = 0.28). In the synbiotic group, SOD activity showed a nonsignificant increase from 2.48 ± 0.11 to 2.57 ± 0.16 U/mL (*p* = 0.66). In the female-control group, SOD activity increased, but not significantly (*p* = 0.13), from 1.28 ± 0.23 U/mL to 1.74 ± 0.12 U/mL. In the female-synbiotic group, an insignificant rise in SOD activity was observed from 2.41 ± 0.15 U/mL to 2.45 ± 0.19 U/mL following synbiotic administration (*p* = 0.88). In the male-control group, SOD activity decreased from 1.84 ± 0.19 U/mL to 1.83 ± 0.12 U/mL. In the male-synbiotic group, SOD activity also rose insignificantly from 2.56 ± 0.16 U/mL to 2.70 ± 0.26 U/mL following synbiotic administration (*p* = 0.68) (Figure 3).

### 3.4. Evaluation of GPx Activity

A nonsignificant increase in GPx activity was observed in the control group from 3.57 ± 0.69 nmol/min/mL to 4.14 ± 0.48 nmol/min/mL (*p* = 0.63), while a nonsignificant increase was observed in the synbiotic group from 2.84 ± 0.62 nmol/min/mL to 4.78 ± 0.57 nmol/min/mL (*p* = 0.12). The level of GPx activity increased also in the female-synbiotic and male-synbiotic groups. However, no significant changes were observed.

## 4. Discussion

The present work was carried out on healthy volunteers randomly divided into groups of men and women, which were then subdivided into synbiotic and control groups. The study was not blinded because the control group did not consume any supplements during this time. Similar results, that is, that probiotic supplementation led to an increase in plasma antioxidant levels, were reported by Martarelli et al. [21] in a study of healthy athletes during intense exercise training.

This paper shows that administration of synbiotics containing* Lactobacillus casei* with Inulin caused a significant increase in the FRAP values of human plasma. FRAP is a sensitive indicator of the total antioxidant status of biological fluid. Many authors [22–27] have reported that probiotics have antioxidant properties, with or without prebiotics. These works indicate that a limited number of probiotic strains may lessen oxidative stress by several mechanisms. Firstly, direct neutralization of oxidants in the intestinal tract is possible by the expression of antioxidant enzymes (e.g., SOD, CAT, and GPx). In addition, stimulation of the immune system can reduce inflammation and prevent cytokine-induced oxidative stress. Another route is by the inhibition of intestinal pathogens, which reduces inflammation and associated oxidative damage. Probiotics may also enhance the absorption of micro- and macronutrients, including antioxidants, or finally reduce postprandial lipids, which are connected with oxidative damage and are often responsible for some food-related pathologies [21, 25].

As mentioned above, Martarelli et al. [21] report that probiotic supplementation (*Lactobacillus paracasei* IMC 502 and* Lactobacillus rhamnosus* IMC 501) led to an increase in plasma antioxidant levels in a group of athletes during four-week intense physical activity. The antioxidant properties of probiotics in human volunteers have been confirmed elsewhere [26]. The antioxidative potential of* L. fermentum* ME-3, examined by blood total antioxidative activity (TAA) and total antioxidative status (TAS), was found to be much higher in the study group than an accompanying placebo group. Probiotics were also examined* in vitro* to investigate their antioxidant properties. This experiment provided an opportunity for a very extensive analysis, which included a range of parameters and a number of bacterial strains.

Amaretti et al. [28] examined several parameters, including ascorbate autoxidation (TAA_AA_) and SOD activity, to determine the antioxidant properties of 34 strains of lactic acid bacteria: 7 species of the* Bifidobacterium* genus, 6 of* Lactococcus*, 10 of* S. thermophilus*, and 11 of* Lactobacillus*. This analysis revealed that the level and particular mechanisms of antioxidant activity are specific for bacterial strain:* Lactococcus* strains were characterized by the highest SOD activity, while* Lactobacillus spp*. and* S. thermophilus* showed highest TAA_AA_. The probiotic strains administered together (DOXO-induced oxidative stress in the animal model) increased the plasma total antioxidant activity of probiotic group. Similarly, Hathout et al. [29] have reported that administration of* Lactobacillus casei* and* Lactobacillus reuteri* increased total antioxidant capacity (TAC) in supplemented rats.

Recently, Gagnon et al. [30] evaluated the bioaccessibility of antioxidants in milk fermented by selected* B. longum* subsp.* longum* strains during* in vitro* dynamic digestion. All strains appeared to have some antioxidant properties evaluated by Oxygen Radical Absorbance Capacity (ORAC) Assay.

Our results show that administration of synbiotics resulted in a significant increase in the CAT activity of human plasma. These findings are consistent with recent studies. Shen et al. [31] demonstrate that CAT activity significantly increases in serum after administration of* B. animalis* 01 ICFE. Chamari et al. [32] report a significant increase in CAT activity in a group of healthy female subjects receiving probiotic supplementation compared to controls. Similarly, Shen et al. [33] demonstrate that* L. plantarum* administration increased CAT activity in liver chickens. Other studies have also shown that probiotics can enhance the activity of CAT in different tissues [34, 35].

Our results indicate that the CAT level was two times higher in women than in men after synbiotic administration. Some studies note that, apart from genetic factors, catalase activities are influenced by a number of environmental factors, for example, seasonal variations [36, 37]. In addition, melatonin can regulate its activity. Its concentration during the day is higher in autumn and winter [38]. Ito et al. [39] suggest that daytime melatonin levels are higher in females with psychological stress derived from living environment, lifestyle habits, or premenstrual syndrome. This observed gender variation in CAT activity could be explained by different levels of hormones, such as testosterone and oestradiol. Testosterone has prooxidant properties while oestradiol acts as an antioxidant. Schröder et al. [40] also confirmed that estrogens have* in vitro* and* in vivo* antioxidant effects.

Some studies have confirmed that sports have an effect on catalase activity. Balog et al. [36] found women to have higher CAT activity than men. However, while sports had no effect on the group of women, sports decreased CAT activity in the autumn in the group of men. It was also observed that the inflammatory diseases are preceded by increased catalase activity in plasma [41–43].

Although our study found that synbiotic administration caused an insignificant increase in SOD activity in human plasma, other authors report a statistically significant increase in activity. These differences can be accounted for by the use of different bacterial strains, doses, and timing of administration. Moreover, in the present study, the subjects were administrated probiotic with prebiotic Inulin and no placebo group was used. Gao et al. [44] note that* Lactobacillus plantarum* FC225 (isolated from fermented cabbages) therapy could significantly elevate the activity of SOD. Wang et al. [45] report that, after application of* L. fermentum *to pigs, the activity of SOD significantly increased in serum. Ejtahed et al. [46] discuss the influence of probiotic consumption on antioxidant status of blood in patients with type 2 diabetes. Probiotic yogurt significantly increased SOD activity in comparison to controls. Another study has also reported increases in SOD activity after synbiotic administration [31].

The present work shows that administration of synbiotics resulted in an insignificant increase in GPx activity in human plasma. However, other studies report significant increases in activity, but these used different strains of probiotics without Inulin. A recent study by Gan et al. [47] notes that selenium-enriched probiotics improve the antioxidant status of piglets and increase blood GPx activity. Pandey et al. [48] report that probiotic* Escherichia coli* CFR 16 ameliorates 1,2-dimethylhydrazine-induced oxidative damage in the colon and liver of rats, by pyrroloquinoline quinone (PQQ) production. The probiotic increased GPx activities to normal levels in the liver and colonic tissues of tested animals. Ghoneim and Moselhy [49] confirm that GPx activity was significantly higher in the tissues of animals fed with probiotics. GPx activity was elevated in the hepatic and muscular microsomes of animals treated with probiotic while it was insignificantly increased in the renal microsomes. Some other works confirm increased GPx activity after administration of probiotics [31, 33, 45, 46].

Furthermore, a significant increase in the activity of antioxidant enzymes (CAT, SOD, and GPx) was observed 90 days after application of* Lactobacillus casei* spp. but without Inulin [50]. However, some studies report decreased antioxidant enzyme activity in various tissues and blood after the application of probiotics. Fabian and Elmadfa [51] note that the mean activities of CAT and GPx decreased significantly in the plasma of healthy women after using probiotics. Vaghef-Mehrabany et al. [52] report insignificant differences in CAT activity and reduced GPx and SOD activity in the* Lactobacillus casei* 01 group. However, the key difference between our study and that of Vaghef-Mehrabany et al. is that their experiment was carried out only on patients with rheumatoid arthritis: an autoimmune inflammatory disease that causes great pain and disability and increasing oxidative stress. Other authors [53] note that lactic acid bacteria (LAB) show no significant antioxidant capacity. Chauhan et al. [54] report that administration of* L. fermentum* Lf1 did not result in any significant change with regard to catalase activity. Cecchi et al. [55] assert that antioxidant activity cannot be regarded as a feature of probiotics, as this characteristic is observed in microorganisms, even potentially pathogenic ones.

In this study, the administration of synbiotic resulted in higher activity of CAT, SOD, and GPx, which are important antioxidant enzymes in the human body. Additionally, FRAP assay of plasma found that total antioxidant capacity increased in the study group compared to the control group after administration of synbiotics. This may indicate that administration of synbiotics resulted in the development of antioxidant defense against the generation of reactive oxygen species.

Many authors describe correlations between probiotics and oxidative stress parameters. Ishii et al. [56] report that* B. breve Yakult* (BBY) oral administration attenuated UV-induced barrier perturbation and oxidative stress of the skin and attribute this antioxidative effect to the prevention of reactive oxygen species generation. Other studies [57, 58] have reported significant decreases in plasma MDA as an effect of bioactive peptides, which are released during fermentation by proteolytic LAB and which are responsible for the beneficial properties of fermented milk products.

Most of identified bioactive peptides were casein derived and they are able to inhibit both enzymatic lipid peroxidation and nonenzymatic lipid peroxidation and exhibit free radical scavenging properties [59]. Additionally, antioxidant peptides are probably related to cysteine-rich proteins that help with glutathione synthesis [60]. A previous study found that while two types of fermented yogurt exhibited some antioxidant activity, the probiotic yogurt was more effective than the conventional yogurt [46]. Several* in vitro* studies confirm the ability of LAB strains to inactivate ROS via enzymatic mechanisms based on a coupled NADH oxidase/peroxidase system. Lin and Chang [61] report that strains of* B. longum* and* L. acidophilus* manifest antioxidative activity and inhibit linoleic acid peroxidation, and this inhibition plays a role in the lipid peroxidation process. Many other works describe the beneficial influence of LAB on the reduction of oxidative damage [62, 63]. Yadav et al. [34] note that probiotics (*Lactobacillus acidophilus* NCDC14 and* Lactobacillus casei* NCDC19) significantly suppress STZ-induced oxidative damage in pancreatic tissues by inhibiting lipid peroxidation (LO) and preserving the antioxidant pool, for example, the activities of SOD, CAT, and GPx.

Recent reports [64, 65] have shown that* S. boulardii* is able to reduce drug induced oxidative damage, especially DNA damage within the pancreatic acinar cells of ANP-induced rats (acute necrotizing pancreatitis). Suryavanshi et al. [66] note that* S. boulardii* extract has the ability to significantly reduce ROS formation in an A549 cell model. The authors [67, 68] also report that homofermentative lactobacilli exhibit high antioxidant activity, while the antioxidant properties of heterofermentative lactobacilli are highly strain-dependent. The incorporation of green tea in a dose-dependent manner can increase the antiradical and ferric reducing ability of probiotics with both bioyogurts and acidophilus milk [69]. Grompone et al. [70] examined 78 strains of* Lactobacillus* and* Bifidobacterium*. Probiotic strains were administered to* C. elegans*;* L. rhamnosus* CNCM I-3690 demonstrated the highest antioxidant capacity. In another study [71] examining 13 strains of* Lactobacillus* spp., maximum antioxidant capacity, measured as oxidation inhibition, was observed in* L. casei *ssp.* casei* 19.

In addition to the production of antioxidants and free radical scavenging substances, probiotics exhibit some metal chelating activities. As transition metal ions can easily initiate LO or initiate the decomposition of hydroperoxides to peroxyl and alkyoxyl radicals, metal chelation influences the antioxidant properties of probiotics [72]. Lee et al. [73] confirmed that the antioxidant activity of* L. casei* KCTC 3260 is connected with the chelation of metal ions. Other studies report that the lactobacilli are able to prevent the production of OH^•^ by the chelation of “free” ferrous ions, which initiate Fenton's reaction [74], and the scavenging of Mn^2+^ [62]. The antioxidative effect of some probiotic strains is highlighted by the inhibition of ascorbate autoxidation, scavenging of free radicals, OH^•^, and metal ion chelation [46, 61, 75].

Although many authors discuss the antioxidant properties of probiotics, the mechanism of their action is not fully understood. Exopolysaccharides released by probiotic bacteria may potentially play a role in reducing oxidative stress. These long-chain polysaccharides consist of branched, repeating sugar units which protect probiotics under starvation conditions or extreme temperature and pH. Kodali and Sen [76] note that* Bacillus coagulans* RK-02 was able to perform extracellular biosynthesis of exopolysaccharides and found that EPS exhibited significant antioxidant activities* in vitro*, when compared to vitamin E and vitamin C. Şengül et al. [77] report lower levels of the oxidative biomarker myeloperoxidase (MPO) in a rat model treated with the low-EPS producing* Lactobacillus* strain A13 than a similar model treated with high-EPS producing* Lactobacillus delbrueckii* ssp.* bulgaricus* B3 following the induction of oxidative stress.

Moreover,* Lactococcus lactis* and* Lactobacillus plantarum* engineered to produce and release SOD demonstrated anti-inflammatory effects in the TNBS colitis model and gave significantly better results than infusion of bovine SOD [78]. Similarly, manganese SOD producing* Lactobacillus gasseri* demonstrated significant anti-inflammatory activity in IL-10-deficient mice [79]. Also, Kullisaar et al. and Chang and Hassan [80, 81] have reported that* Streptococcus thermophilus* and* Lactobacillus fermentum* strains show significant antioxidative activity thanks to production of SOD. Similarly, de Moreno de LeBlanc et al. [82] confirm that a CAT-producing* L. lactis* strain was able to prevent the occurrence of tumors in an experimental DMH-induced colon cancer model. Elsewhere [83], it was found that the cooperation between CAT and SOD could significantly enhance oxidative resistance in* L. rhamnosus*.

Some authors have described the implication of probiotic use in the prevention and treatment of various diseases. Some refer to the antioxidant hypothesis when discussing cardiovascular disease (CVD) prevention and treatment using* Lactobacillus strains*. Wang et al. [84] have proved that* L. plantarum* CAI6 and* L. plantarum* SC4 may protect against CVD by lipid metabolism regulation and Nrf2-induced antioxidative defense in hyperlipidemic mice. Lutgendorff et al. [85] report that probiotic pretreatment could successfully prevent acute pancreatitis- (AP-) induced GSH depletion, stimulate mucosal GSH biosynthesis compared with control and sham-operated rats, and prevent moderate AP-induced barrier dysfunction. van Minnen et al. [86] note that pretreatment with these probiotics attenuated bacterial translocation and resulted in reduced experimental mortality in AP rats.

## 5. Conclusions

Our results demonstrate that synbiotics containing* L. casei* with Inulin are effective compounds that protect the human body from oxidative stress damage. Synbiotics may have a positive influence on blood plasma antioxidant capacity and the activity of selected antioxidant enzymes, such as CAT. Therefore, the described synbiotic might be considered as a food supplement suitable for the prevention and treatment of oxidative stress injury.

## Abbreviations

CAT: Catalase
CFU: Colony-forming unit
EDTA: Ethylenediaminetetraacetic acid
FRAP: Ferric reducing ability of plasma
GPx: Glutathione peroxidase
GR: Glutathione reductase
GSH: Reduced glutathione
GSSG: Glutathione disulfide
LAB: Lactic acid bacteria
MDA: Malondialdehyde
NADPH: Nicotinamide adenine dinucleotide phosphate
ROS: Reactive oxygen species
SOD: Superoxide dismutase
TAA: Total antioxidative activity
TAC: Total antioxidant capacity
TAS: Total antioxidative status
GSHt: Total glutathione
TPTZ: 2,3,5-Triphenyltetrazolium chloride.
